# Medical Students’ Attitudes and Beliefs towards Psychotherapy: A Mixed Research Methods Study

**DOI:** 10.3390/bs7030055

**Published:** 2017-08-18

**Authors:** Costas S. Constantinou, Maria Georgiou, Maria Perdikogianni

**Affiliations:** 1 Medical School, University of Nicosia, 46 Makedonitissas Ave, Nicosia 1700, Cyprus; perdikogianni.m@unic.ac.cy; 2 Faculty of Social Sciences, University of Nicosia, 46 Makedonitissas Ave, Nicosia 1700, Cyprus; georgiou.mar@unic.ac.cy

**Keywords:** attitudes toward psychotherapy, medical students, mixed research method

## Abstract

*Background*: Research findings suggest that attitudes towards psychotherapy predict willingness to seek therapy. However, understanding how medical students think about using and referring their patients for psychotherapy is limited. *Aims*: The aims of this study are to measure medical students’ attitudes towards professional help seeking, and to investigate the reasons for whether or not they would refer their patients to psychotherapy in their future role as doctors. *Method*: The participants were 127 medical students in their first and second year of the MBBS4 programme at the Cyprus campus of St George’s University of London, who completed a self-report measure of attitudes towards psychotherapy and a semi-structured interview. *Findings*: Participants showed general positive attitudes towards psychotherapy, but were reluctant to use or refer their patients, largely due to perceived stigma and accessibility. *Conclusions*: Medical students should be further trained in order to become more confident in using psychotherapy and referring their patients.

## 1. Introduction

A number of research studies have provided evidence supporting the effectiveness of psychotherapy in the treatment of several mental health disorders. Psychotherapy has proved effective in treating people diagnosed with anxiety disorders such as obsessive compulsive disorder (OCD), post-traumatic stress disorder (PTSD), and panic attacks [1,2,3,4,5,6]. Some studies suggest that psychotherapy is as effective as psychotropic medication in treating disorders such as depression and anxiety [7]. 

Interestingly, attitudes towards psychotherapy seem to influence the willingness of an individual to seek or not seek professional help. That is, individuals who perceive professional help seeking in a positive respect are more likely to seek mental health treatment than those that have a negative attitude towards it [8,9]. A qualitative study by Hill et al. [10] showed some interesting results regarding the positive attitudes of university students towards psychotherapy. The participants in her study believed that psychotherapy should be sought out when people have issues they cannot handle on their own, or when they suffer from serious conditions such as depression and anorexia. The same participants perceived psychotherapy as being very beneficial in the treatment of various mental health problems, especially with conditions such as depression and anorexia. 

In addition to negative attitudes affecting individuals’ responses to psychological treatment, factors such as past experience with psychotherapy and racial differences may also influence decisions to seek psychological help. Certain studies have examined the role of past experiences with psychotherapy and racial differences as factors that impact the attitudes of college students towards seeking professional psychological help. Masuda et al. [11] found that positive past experiences with psychotherapy predicted favorable attitudes towards help seeking. In another study, Masuda et al. [12] showed that racial differences exist in relation to attitudes towards seeking professional psychological help, as results indicated that Asian American and African American college students were less likely to seek professional help than European American college students. 

Although individuals may acknowledge the benefits of psychotherapy, certain barriers may prevent them from seeking professional help. The most common barrier to seeking psychotherapy seems to be public and self-stigma [13]. So et al. [14] explained that stigma and self-concealment were negatively associated with positive help-seeking attitudes in college students. Other barriers include the beliefs that one’s problems are not serious enough to justify seeking professional help [15], and that the cost is more than the benefit [16]. Fear of judgment, ignorance about the nature of psychotherapy and negative attitudes towards discussing painful emotions were reported as additional barriers to seeking psychotherapy [17,18,19]. Upon looking at university students’ attitudes, Hill et al. [10] found that perceived stigma and breach of confidentiality were reported as important barriers to seeking therapy, while cost and disbelief in the effectiveness and necessity of psychotherapy were found to prevent attempts to seek professional help [10].

Despite the wealth of studies on the general college population regarding attitudes towards psychotherapy, little is known about the attitudes of medical students. It has been documented that medical students experience high levels of stress and anxiety [20,21] during their studies, and it would be interesting to examine their attitudes towards seeking help for themselves, as well as their willingness to refer their patients for psychotherapy as future physicians. Very few studies have attempted to investigate medical students’ attitudes towards psychotherapy. Voracek et al. [22] examined the attitudes of 150 medical students using an indirect measure of attitudes before and after a short interval of psychotherapeutic intervention. The study revealed that the students’ predominantly positive attitudes towards psychotherapy were enhanced after the completion of the course of therapy, which indicated the impact of such an intervention on students’ attitudes. Ey et al. [23] conducted a survey study to examine the willingness of medical trainees to participate in a resident wellness program that offered counseling services on site. The results showed that reluctance to use the service was associated with concerns about confidentiality, the helpfulness of counseling, stigma, and lack of time. In another study by Brimstone et al. [24], 132 medical students were asked to score 16 statements relating to concerns about seeking mental health care after reading a vignette about a student that experienced depression. The results showed that medical students were more likely to seek advice from friends and family when faced with a problem, while barriers to seeking professional help were related to issues of confidentiality and cost. 

An in-depth understanding of medical students’ attitudes towards psychotherapy is very important, because as future practitioners, they might come across patients who would benefit from professional psychological support. Studies show that patients with a chronic disease are more likely to adhere to their prescribed therapy and eventually achieve positive health outcomes when they receive psychotherapy or other psychological support. More specifically, although von Wietersheim and Kessler [25] did not indicate a direct positive effect of psychotherapy on the course of chronic inflammatory bowel disease, they did find that psychotherapy improved patients’ psychological well-being and their overall self-management of the condition. A meta-analysis of literature between 1948 and 2001 by DiMatteo [26] revealed a strong association between psychosocial support and adherence to medical treatment. Along similar lines, Ogden [27] presented a series of studies that showed a link between stress and risky behaviour, and highlighted the importance of psychological support and stress management interventions. Therefore, improving medical students’ attitudes towards psychotherapy can potentially encourage them to increase the referrals of their patients for psychological support when such support is needed once they are practicing doctors. Furthermore, understanding the barriers to using or referring patients to psychotherapy could lead to taking action in tackling such barriers and consequently improving patients’ psychological well-being, treatment adherence, and overall health outcomes. 

The existing research in understanding medical students’ attitudes towards psychotherapy is limited. In order to fill this gap, we aimed to use a mixed research methodology, so as to achieve a deeper understanding of medical students’ attitudes towards psychotherapy treatment. For the quantitative part, we used a standardized and reliable measure: the short version of the Attitudes Toward Seeking Professional Psychological Help-Scale [28] in order to assess the attitudes of medical students towards psychotherapy. We also examined the relationship between attitudes and various factors such as gender, nationality, and prior experience with psychotherapy. In order to achieve a more in-depth understanding of the reasons why medical students feel either positively or negatively toward psychotherapy, we also conducted qualitative interviews [29]. For the interviews, we used a semi-structured questionnaire based on the one used by Hill et al. [10], which aimed to assess the beliefs of medical students about their willingness to seek therapy for themselves and refer their patients for therapy as future doctors.

## 2. Methodology

### 2.1. Participants

The sample consisted of 127 medical students in their first and second year of the MBBS4 programme of St George’s, University of London Medical Programe at the University of Nicosia Medical School. Fifty-nine participants were female (46%) and 67 were male (52%). Participants were between 20 and 54 years of age, with a mean age of 25.01. One participant did not fill in demographic data regarding gender. An ethnic classification (White/Non-Hispanic, Black/African-American, Latino, Asian, Mixed ethnic background) that is widely used in the United States was adapted for the study, and the results yielded participants from four ethnic groups: 68 Whites, 9 Black/African-Americans, 21 Asians, and seven from a mixed ethnic group. Twenty-three participants did not state their nationality. Twenty-five participants reported having had psychotherapy in the past, and 102 stated that they had never received any form of psychotherapy. From this sample (127 medical students), 12 participants were interviewed using the semi-structured questionnaire. The participants were first and second year students who did not have any specialised training in psychotherapy. However, they had to undertake 30 h of lectures in sociology and psychology, and a 30-h course in communication skills. During those sessions, participants were informed about the different types of psychological interventions and their effectiveness, and they were specifically taught about the use of cognitive behavioural therapy (CBT) and how it can be applied to patients with anxiety disorders. For example, in one of these sessions during their second year of studies, students learned how they could explain CBT to a patient and discuss its effectiveness with them. This enabled patients to reflect on situations where they had to cope with and manage stress in their lives, as well as understand somatic symptoms related to anxiety disorders such as pain, palpitations, or diarrhoea.

### 2.2. Measures

#### The Attitudes toward Seeking Professional Psychological Help-Scale [28] 

The ATSPPH-S was originally developed in 1970, and revised in 1995 by Fischer and Farina. The updated version is a shortened 10-item scale that produces a single score representing the respondent’s core attitude toward seeking professional psychological help. According to Fischer and Farina [28], the internal reliability of the ATSPPH-S is 0.84, and the test-retest reliability at four weeks is 0.80.

The items are rated on a four-point Likert scale (0 = disagree, 1 = partly disagree, 2 = partly agree and 3 = agree) for items 1, 3, 5, 6, 7. Items 2, 4, 8, 9, 10 are reversely scored (3 = disagree, 2 = partly disagree, 1 = partly agree and 0 = agree). Scores range between 0 and 30. A high score indicates a more positive attitude toward seeking psychological help. Internal consistency and reliability have been found to be adequate for this scale [16,28]. Total scores of 13 or higher indicate positive attitudes toward seeking professional psychological help, while scores of 12 or lower indicate negative attitudes.

#### Semi-Structured Interview Questionnaire

A semi-structured interview questionnaire based on the interview protocol used by Hill et al. [10] was used to record beliefs and assess attitudes about psychotherapy. Permission was obtained from Dr. Hill to use this questionnaire in our study. The questionnaire included the following questions:

1. What comes to mind when you think about psychotherapy? 2. What are your beliefs about psychotherapy? 3. How did you develop these beliefs about psychotherapy? 4. When should a person seek psychotherapy? 5. At what point would you personally consider seeking psychotherapy for yourself? 6. Under what circumstances would you refer a patient for psychotherapy? 7. What is the client’s role in psychotherapy? 8. What is the therapist’s role in psychotherapy? 9. What are the benefits of seeking psychotherapy? 10. What are the barriers to seeking psychotherapy? 11. What would prevent you from referring a patient for psychotherapy? For the purposes of this paper, we focused on the results that related largely to the reasons why medical students felt positive about psychotherapy, and what they perceived as barriers to seeking professional help. 

### 2.3. Procedure

Permission to carry out this study was granted by the Research Ethics Committee of the University of Nicosia, where the study took place, and by the Cyprus National Bioethics Committee. A convenience sampling method was used to recruit medical students studying at the University on a volunteer basis. The students were told that the study would examine the attitudes of medical students toward seeking professional psychological help. The questionnaires were distributed to the entire year 1 and 2 cohorts, who were asked to complete them anonymously and return them to the researchers. For the semi-structured interviews, students were informed about the study by email, and those who expressed an interest in participating were given an appointment and interviewed by one of the researchers. The interviews took a maximum of 30 min each, and were audio recorded. 

### 2.4. Data Analyses

The coding and analysis of ATSPPH-S was done through SPSS, which helped us measure the mean score of the participants, and show whether the score was positive or negative towards psychotherapy. A correlation between the score and a number of participants’ characteristics such as gender, nationality and prior experience with psychotherapy was also investigated. 

**Figure 1:**
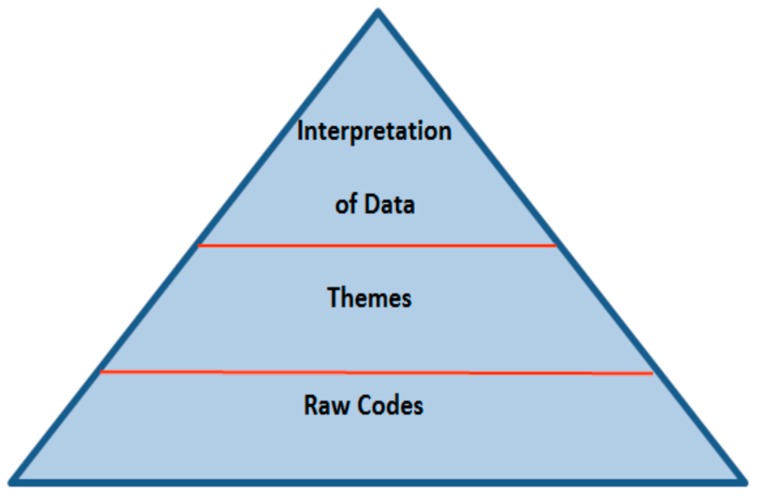
Pyramid of coding and analysis.

All interviews were transcribed verbatim. The study relied on both deductive and inductive approaches. The deductive part consisted of four pre-determined areas of inquiry, namely: understanding of psychotherapy, use, barriers, and the roles of clients and therapists. Within these four areas, we used a “general inductive approach” [30] and generated codes directly from the data. Thomas [30] (p. 242) described the process of inductive coding as follows: “label the segments of texts to create categories (30–40 categories) => reduce overlap and redundancy among categories (15–20 categories) => create a model incorporating most important categories (3–8 categories)”. Based on Thomas’ [30] approach, we used a pyramid of coding and analysis by initially generating raw codes, and then themes (Figure 1). Raw codes were keywords that came out from participant’s words, while themes represented raw codes that fell under similar categories. To ensure the quality of coding, we adopted a double-blind procedure for coding by having two researchers generate raw codes and themes independently [31]. The two researchers then met and reviewed the material and the codes in order to refine and finalise them. Based on themes that were generated, we proceeded with the description and interpretation of the data. The pyramid approach shows that all of the codes derived from the basis (data) and its volume were narrowed down through group coding, identification of themes, and interpretation. The sample size was determined at two levels. We initially sampled 10 interviews, based on Francis et al.’s [32] recommendation. As two more students expressed an interest in participating, we decided to include those interviews in our initial sampling. 

We created a codebook on the basis of the pyramid above. In the first layer of the pyramid, we identified 401 raw codes, which directly related to our research question. We then identified codes that fell under similar areas and ended up with 20 themes (second layer). We used these 20 themes in order to interpret the data by addressing our research question and the areas of inquiry. To ensure that we had enough data for analysis, we checked for data saturation [33]. We measured all themes for the first interview, and then proceeded to measure the shared themes between the first and second interview, and identified any new themes from the second interview. We followed the same process with all of the interviews. The purpose of this procedure was to identify the moment when no new themes derived from the data. The number of themes was zero at the fifth interview, and continued to be so until the twelfth interview. Based on the above, we safely concluded that we had reached data saturation and we decided to include all interviews (not only the first five ones) in the analysis. 

## 3. Results

### 3.1. Quantitative Results

Descriptive statistics showed that the mean attitude score was 17.2, which indicated positive attitudes. Independent-samples t-tests were conducted to compare the attitudes scores of males and females. As per Table 1 below, the results showed that there was no significant difference in scores for males (*M* = 17.01, *SD* = 5.94) and females, (*M* = 17.49, *SD* = 5.88); *t* (124) = 0.45, *p* = 0.65. 

With regard to nationality (Table 2), there was no significant difference between Black (*M* = 16.22, *SD* = 5.72) and Asian students (*M* = 16.76, *SD* = 7.42); *t* (28) = −19, *p* = 0.85, and between Black (*M* = 16.22, *SD* = 5.72) and Mixed race students *(M* = 11.57, *SD* = 4.20); *t* (14) = 1.80, *p* = 0.09. The difference between Asian (*M* = 16.76, *SD* = 7.42) and Mixed race students *(M* = 11.57, *SD* = 4.20); *t* = (26) = 1.74, *p* = 0.09 did not show statistical significance. Furthermore, there was no statistically significant difference found between White (*M* = 17.64, *SD* = 5.49) and Black students (*M* = 16.22, *SD* = 5.72); *t* = (75) = 0.73, *p* = 0.47, and between Asian ((*M* = 16.76, *SD* = 7.42) and White participants (*M* = 17.64, *SD* = 5.49); *t* = (86) = 0.65, *p* = 0.52. However, White (*M* = 17.64, *SD* = 5.49) participants were more positive than Mixed race participants *(M* = 11.57, *SD* = 4.20); *t* = (72) = 2.88, *p* = 0.005, and this was statistically significant.

Finally, as per Table 3 below, the results showed a significant positive correlation between those who used psychotherapy in the past (*M* = 20.64, *SD* = 4.93) and those who did not use it in the past (*M* = 16.48, *SD* = 5.85); *t* = (125) = 3.28, *p* = 0.001.

### 3.2. Qualitative Results

In this part, we analyzed the results from the qualitative interviews in order to shed light on medical students’ attitudes towards psychotherapy. We focused on medical students’ beliefs about psychotherapy, their willingness to use psychotherapy or refer their patients for it, and the reasons for not using psychotherapy.

#### 3.2.1. Why are Medical Students Generally Positive toward Psychotherapy?

##### Medical Students’ Beliefs about Psychotherapy

Participants used an utilization approach when they expressed their beliefs about psychotherapy. All participants had a positive belief about psychotherapy in terms of its utility, as they perceived it as helpful for people in need and part of a more holistic understanding and intervention. More specifically, a participant said:

*“I support* *it, I think everyone should have it, but not necessarily everyone has to do it because a lot of people have their coping mechanisms, which are either their friends or families, but not all people have that—ability to talk to someone. So, in some cases and especially in very traumatic cases, or if someone has very complicated problems, it’s best probably addressed by someone who is professional and knows what they are doing.”*

Overall, participants would recommend psychotherapy to someone they thought would benefit from it. Participants’ beliefs came from a variety of sources, but could be organized into two main areas. First, participants had institutionally-induced beliefs. That is, they had beliefs that were influenced by the institutions they were socialized in, such as education and the media. Second, participants’ beliefs came from their own experience, whether personal or that of other close people’s experiences, such as friends and family. Interestingly, although all participants expressed positive views towards psychotherapy, there was a difference between those who used psychotherapy in the past and those who did not in terms of the words they used to describe it. The participants who used psychotherapy in the past used adjectives that added more value to its mere utility, such as “great”, “wonderful”, and “powerful”. The participants who did not use psychotherapy in the past described it merely in terms of its usefulness and used words and phrases such as “helpful”, “useful”, and “treatment tool”.

##### Use of Psychotherapy

Participants were asked to express their views about the use of psychotherapy for someone else and themselves, as well as explain what the benefits were and under what circumstances they would refer a patient for psychotherapy. Participants’ views about the use of psychotherapy aligned with their positive beliefs. That is, they maintained that someone (including themselves) would use psychotherapy when they had a disrupting experience in their life or when they were encouraged to do so by other people, such as family members. Thereafter, psychotherapy would be a useful tool for re-establishing their psychological well-being. In addition, psychotherapy would not only make them feel better psychologically, it would also help them regain their position in society, such as being productive and effective in the workplace, at home, and among their peers. The use of psychotherapy, interestingly, would mean the re-institutionalization of the individual—that is, the re-integration of the individual in social institutions. For these reasons, participants would generally refer their patients for psychotherapy. Some participants explained that another reason for seeking professional help would be an individual’s decision not to talk to friends or family about their problems, and seek professional help as a result.

To support their views about the benefits of psychotherapy, participants used a generalization and specification approach. More specifically, they generalized the benefits from psychotherapy based on the knowledge they acquired from their studies and the media, and they referred to specific cases that involved their friends or family members. 

Interestingly, participants understood the use of psychotherapy as a tool for the management of a problem, but not for preventive purposes or for personal development. For example, they would not think to use psychotherapy themselves or refer a patient for psychotherapy when they experienced difficulties in their life that they thought they could handle, before developing any psychological disorders. This finding possibly accorded with participants’ preoccupation with a possible stigma (see next sub-section for more information) that might attach to someone who is seeking professional help for psychological problems. That is, they would seek help only when there was no other way out. In that case, getting well would be more important than being stigmatized. 

#### 3.2.2. What Could Have Caused Medical Students to Opt Out of Psychotherapy?

The main barrier for not using psychotherapy, which was expressed by all participants, was stigma. Participants explained that the fear of being stigmatized as “crazy” would be an important obstacle for someone or themselves not to use psychotherapy, or not to refer their patients for it. For example, a participant said:

*“The* *stigma that exists in psychotherapy and all the treatments related to psychiatric illnesses is really important”.*

The stigma was mentioned by almost all participants as a strong barrier. Another participant was even more specific:

*“One* *is just the social barrier of ‘I’m not crazy, I don’t wanna go and see a psychotherapist, I’m not crazy, I’m not that person’”.*

Another perceived barrier was accessibility. To participants, inaccessibility meant two things: the financial cost of psychotherapy, and second, a lack of knowledge about the usefulness of psychotherapy. 

**Figure 2:**
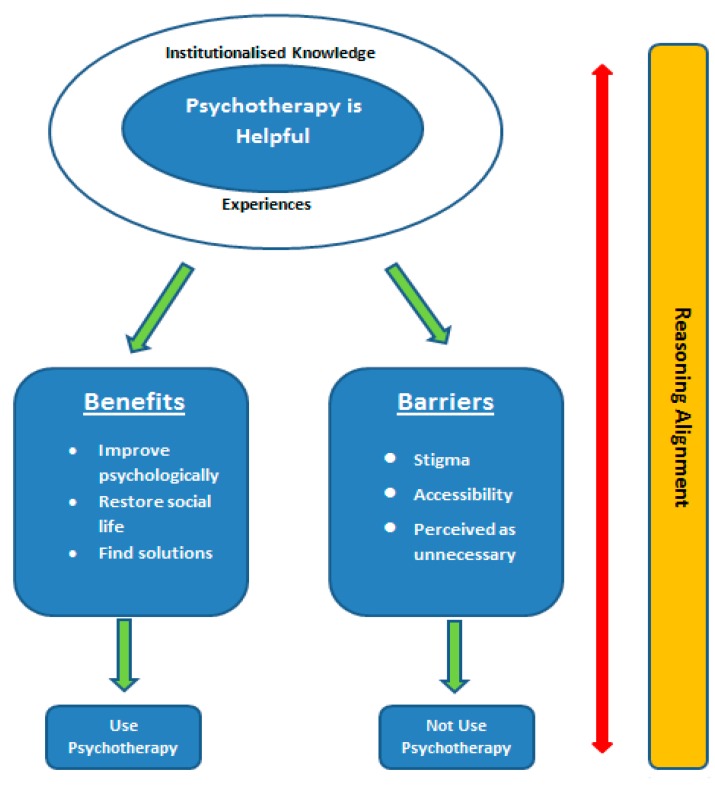
Participants’ understanding of and attitudes towards psychotherapy.

The barriers that prevented medical students to seek out psychotherapy support for themselves, as mentioned above, were the same barriers that would prevent them from referring patients for such support as future doctors. If an individual perceived psychotherapy as a stigma for themselves, then they perceived it as a stigma for others as well, and would thus refrain from referring others to this kind of support. Other reasons participants would not refer patients for psychotherapy included their belief that psychotherapy would potentially culminate in other problems or difficulties. In addition, participants would not refer their patients to a psychotherapist if the patients were not be willing to have an active role in the psychotherapy process, or could solve their problems themselves. Based on their previous views on benefits, participants used what we call “a reverse reasoning alignment”; that is, they would not refer their patients for psychotherapy if the reasons for referring (benefits, willingness, etc.) were not present or were reversed. Figure 2 shows the reasoning and reverse reasoning as constructed by the participants of this study. 

## 4. Discussion

One of the aims of the study was to assess medical students’ attitudes towards professional help seeking using a quantitative standardized measure. The findings from the quantitative data reveal that medical students have positive attitudes towards psychotherapy. No difference was found between males and females. However, there was a difference between white and mixed race participants, with white participants showing more positive attitudes than the mixed race group. This supports research findings by Masuda [20] that proposed ethnic differences in attitudes towards professional help seeking. A possible explanation why white people are more positive to seeking psychotherapy support than a mixed race population may be due to the cultural perception of the use and effectiveness of psychotherapy. For example, Kleinman [34] explained that the word “depression” is not well received by Chinese people, because they associate it with too much anger and negative thinking. Instead, Chinese people tend to express more physical symptoms than psychological ones and prefer the word “neurasthenia”, which means to them “neurological weakness” [35] (p. 229). Another finding of this study was that those who received psychotherapy in the past showed more positive attitudes than those that did not, which again supports previous research studies by Masuda [11] that yielded similar results. Prior experience helps because it ameliorates biases and makes those who have tried psychotherapy aware of its usefulness [36].

The results from the qualitative interviews helped explain why medical students were generally positive towards psychotherapy. Medical students expressed positive beliefs because they considered it a tool that people can use to solve a problem they cannot solve on their own. This positive belief derived mainly from their training at a university, the media, and from prior experience with psychotherapy. This finding is in accordance with Hill et al.’s [10] study, which indicated that participants would utilise psychotherapy if they had a disrupting experience that they could not handle on their own. Interestingly, medical students would not use psychotherapy only as a means to solve a problem, but they would use it as a framework for re-integrating themselves into social settings. Furthermore, our study showed that psychotherapy was largely understood as a way to solve problems, rather than a means for self-development and personal growth. Hill et al. [10] found similar results in this respect. This possibly has to do with the association of the use of psychotherapy as an option to address psychological problems and disorders and not as a way to explore feelings, learn to problem solve, and generally achieve emotional self-growth. 

Medical students scored an average of 17.2 in the ATSPPH-S scale, which showed that they were generally positive towards psychotherapy. However, they could have scored much higher. The highest score that could have been reached was 30, given that they had some exposure to the different types of psychotherapy (i.e., CBT) and were educated as to their importance during the first two years of their study. What is not clear from the score itself is why they did not score higher. The qualitative results have been very illuminating. It seems that some of the barriers to using or referring patients for psychotherapy are powerful enough to keep medical students at a distance. The strongest barrier that this study found is that of stigma. Medical students considered a potential stigmatization of being labelled “crazy” as an obstacle either for utilizing psychotherapy themselves, or referring their patients for it. This is in line with other studies, which found that the fear of breaking confidentiality was a serious barrier for their participants [24], and that stigma was an important barrier to seeking psychotherapy [13]. The question is, why is this perceived stigma so powerful? By being stigmatized as “crazy” or “mentally ill”, patients would possibly be perceived by others as having serious issues in their social life or not being stable socially, which in turn would cause them to have problematic social relations. Such stigmatization would hold other people back from socializing with the “stigmatized" person. In other words, being stigmatized as “crazy” may lead to further stigmatization and social isolation, which are understood as important obstacles to seeking psychotherapy. So, the social and psychological costs of seeking psychotherapy are perceived as greater than the benefits. 

Other barriers to seeking psychotherapy were related to issues of accessibility and related to financial cost or lack of knowledge of the benefits of psychotherapy. Participants were also reluctant to refer patients for psychotherapy if they believed that patients would not have an active role in the therapeutic process. Our findings are supported by Vogel et al. [15], who proposed that people would not seek psychotherapy if they thought that they could deal with their problems on their own, while Komiya et al. [18] suggested that ignorance about the nature of psychotherapy is a discouraging factor.

The most important findings from this study that can contribute to the improvement of help-seeking behaviours are that medical students are positive when they have been exposed to psychotherapy themselves, and that they are not willing to either utilize psychotherapy or refer their patients for it when the perceived stigma outweighs the perceived benefits and outcomes. These two main findings can have important future implications, which are discussed below. 

We now know that medical students are generally positive towards psychotherapy, but they may not use it themselves or refer patients for it due to the impact of stigma. Based on this finding, medical students could be trained on two levels. First, they could be trained more thoroughly on the importance of psychotherapy and how it can be used in medical practice. Medical students would then further appreciate the importance of psychotherapy, not only in terms of it helping their patients deal with issues they cannot handle on their own, but also in the sense of doing their job more effectively as doctors, communicating better with their patients, improving their patients’ adherence, and eventually achieving positive health outcomes. The second level on which medical students could be trained involves teaching them ways to override the socially constructed stigma, either for themselves or their patients. By learning that stigma is a purely social product and there is nothing objective behind it, students can understand it as a manageable perception and focus more on the importance and effectiveness of psychotherapy instead. The doctors’ job in handling stigma would be much easier if the general public was informed about its nature, as well as ways to deal with it. Such public awareness and education could have taken place from an early age during school years.

Furthermore, as the financial cost of therapy and the lack of knowledge regarding the usefulness of psychotherapy were mentioned as barriers to seeking help, medical schools could consider providing free counseling services for their students, while health care systems could incorporate free psychotherapy services to patients and their families alongside their other services. 

One of the limitations of this study was the small size of the sample. Participants were also students recruited from year one and two of one specific medical school; therefore, the results are largely indicative of medical students’ attitudes toward psychotherapy. Furthermore, although a statistically significant difference was found between white and mixed race participants, with whites showing more positive attitudes than the mixed race group, this should be treated with a degree of caution since there were only seven students in the mixed race group. The number is too small and these findings need to be replicated in larger samples before firm conclusions can be drawn in relation to differences in attitudes towards professional help seeking between white and mixed race samples.

## 5. Conclusions

This paper discusses the findings of a study of medical students’ attitudes toward psychotherapy in order to fill in the gap that exists in the literature in this area. To ensure adequate and deep understanding of medical students’ attitudes towards psychotherapy, the study relied on a mixed research design. That is, it combined quantitative findings derived from the completion of the validated questionnaire, The Attitudes toward Seeking Professional Psychological Help-Scale (ATSPPH-S) by 127 medical students and qualitative data from 12 in-depth interviews. The results indicated that medical students are generally positive about psychotherapy (score 17.2/30), but perceived barriers prevented them from scoring higher. Though the financial cost of psychotherapy and poor awareness of its benefits are understood as barriers to either using psychotherapy themselves or referring their patients for it, the most important barrier for medical students is perceived stigma and how psychotherapy can portray its user as crazy or insane to the rest of society. Interestingly enough, although the participants had some form of exposure to the different types of psychotherapy and its effectiveness, and had received a lot of information on cognitive behavior therapy (CBT) and how it can benefit certain patients, when asked the hypothetical question of whether they would refer their patients for such support, their response was that they would do so only if the benefits (i.e., psychological improvement, restoration of social life) outweighed the perceived cost from stigma. These research findings highlight the importance of providing further education and exposure about the positive effects of psychotherapy on patients. The results of the study could have been different if the participants were fully acquainted with the concept of psychotherapy and its uses as well as its benefits during their medical school training. Future studies could investigate the impact of knowing about psychotherapy on attitudes towards professional help seeking. 

## Figures and Tables

**Table 1:** *t*-test for gender differences on attitudes towards help seeking.

		N	Mean	SD	*t*	Sig
Gender Differences						
Attitudes toward help seeking	Male	67	17.01	5.94		
					0.45	0.65
	Female	59	17.49	5.88		

*p* < 0.05.

**Table 2:** *t*-test for nationality on attitudes towards help seeking (the table only depicts the groups that showed statistically significant results).

Nationality	N	Mean	SD	*T*	Sig
White	68	17.73	5.49		
				2.88 *	0.005
Mixed	7	11.57	4.2		

* *p* < 0.05.

**Table 3:** *t*-test for prior use of psychotherapy on attitudes towards help seeking.

Use of Psychotherapy	N	Mean	SD	*t*	Sig
Yes	25	20.64	4.93		
				3.28 *	0.001
No	102	16.48	5.85		

* *p* < 0.05.
